# Artificial Intelligence in Relation to Accurate Information and Tasks in Gynecologic Oncology and Clinical Medicine—Dunning–Kruger Effects and Ultracrepidarianism

**DOI:** 10.3390/diagnostics15060735

**Published:** 2025-03-15

**Authors:** Edward J. Pavlik, Jamie Land Woodward, Frank Lawton, Allison L. Swiecki-Sikora, Dharani D. Ramaiah, Taylor A. Rives

**Affiliations:** 1 Division of Gynecologic Oncology, Department of Obstetrics and Gynecology, Chandler Medical Center-Markey Cancer Center, University of Kentucky College of Medicine, Lexington, KY 40536-0293, USAtaylor.rives@uky.edu (T.A.R.); 2 University of Kentucky College of Medicine, Lexington, KY 40536-0293, USA; jamie.land@uky.edu (J.L.W.); ddramaiah1@uky.edu (D.D.R.); 3 SE London Gynecological Cancer Centre, Emeritus Surgeon, London SE5 9RS, UK; fglawtongyn@aol.com

**Keywords:** artificial intelligence, large language models, generative AI, accuracy, chatbot, gynecologic oncology, clinical medicine

## Abstract

Publications on the application of artificial intelligence (AI) to many situations, including those in clinical medicine, created in 2023–2024 are reviewed here. Because of the short time frame covered, here, it is not possible to conduct exhaustive analysis as would be the case in meta-analyses or systematic reviews. Consequently, this literature review presents an examination of narrative AI’s application in relation to contemporary topics related to clinical medicine. The landscape of the findings reviewed here span 254 papers published in 2024 topically reporting on AI in medicine, of which 83 articles are considered in the present review because they contain evidence-based findings. In particular, the types of cases considered deal with AI accuracy in initial differential diagnoses, cancer treatment recommendations, board-style exams, and performance in various clinical tasks, including clinical imaging. Importantly, summaries of the validation techniques used to evaluate AI findings are presented. This review focuses on AIs that have a clinical relevancy evidenced by application and evaluation in clinical publications. This relevancy speaks to both what has been promised and what has been delivered by various AI systems. Readers will be able to understand when generative AI may be expressing views without having the necessary information (ultracrepidarianism) or is responding as if the generative AI had expert knowledge when it does not. A lack of awareness that AIs may deliver inadequate or confabulated information can result in incorrect medical decisions and inappropriate clinical applications (Dunning–Kruger effect). As a result, in certain cases, a generative AI system might underperform and provide results which greatly overestimate any medical or clinical validity.

## 1. Introduction

Over the past two years, artificial intelligence (AI), particularly generative AI (also known as narrative AI), has garnered immense attention. This surge is largely due to the success of combining machine learning algorithms with natural language processing (NLP) and large language models (LLMs), demonstrated through the release of OpenAI’s ChatGPT-3.5 (30 November 2022), ChatGPT-4 (14 March 2023), and ChatGPT-4 Turbo (November 2023) [1]. This led to the opportunity to enter commands or queries in natural language and to receive results in natural language when appropriate. It became possible to enter natural language requests for information, image generation, and even programming. Because of the advances in speech-to-text and text-to-speech technology, it was a short jump from text requests and texts outputs to spoken NLP inputs by humans, as well as NLP outputs in responses that could be both read and listened to. Importantly, the ChatGPT family was developed to sound highly coherent. At present, it is difficult to determine the exact number of generative AI systems that exist because of the rapid ongoing emergence of new models. However, including large-scale models developed by major companies and smaller-scale specialized models used by startups and researchers, there are probably thousands [2], with a curated list current to January 2025 estimating that over 5000 different generative AI model systems exist [3,4]. This large number alone underscores the reality of evolution in generative AI systems—some will differentiate into specific niche utilizations, while others will pursue a singularity, dominating all other forms of intelligence [5].

Google Bard, a generative AI chatbot that was introduced in March of 2023, utilizes its own large language AI model called Gemini [6]. Gemini is able to harvest current information from the Internet and is available in over 40 languages. Microsoft launched Copilot within its Edge browser in February 2023. It uses its own large language model Prometheus, which was built on the Open AI GPT-4 foundation [7].

Highly regarded, comprehensive global reviews of AI and machine learning current to 2023 exist in the literature in relation to conventional and molecular medicine [8,9]. The present review focuses on AI systems that have a clinical relevancy evidenced by application and evaluation in clinical publications. This data-driven relevancy will speak to both what has been promised and what has been delivered by various AI systems.

## 2. Materials and Methods

The resources included in this review were collected monthly from surveys of select journals with high impact factors [10] as part of efforts to summarize significant literature for the International Gynecologic Cancer Society [11,12]. Journals included in this monthly survey include the following: American Journal of Obstetrics and Gynecology, American Journal of Clinical Oncology (ASCO), British Journal of Cancer, Cancer, Cancer Research, Clinical Cancer Research, Gynecologic Oncology, International Journal of Gynecologic Cancer, International Journal of Cancer, Journal of the American Medical Association (JAMA), JAMA AI, JAMA Oncology, Journal of Clinical Oncology, Journal of the National Cancer Institute, Lancet, Lancet Oncology, Molecular Oncology, Nature, Nature Cancer, New England Journal of Medicine (NEJM), NEJM AI, Obstetrics & Gynecology, Oncology, and Proceedings of the National Academy of Science, Science Translational Medicine. This survey also collected literature information through email alerts received from ASCO Journals, Google Scholar Alerts, JAMA Internal Medicine, JAMA Network, JAMA Network Open, JAMA Surgery, JAMA Health Forum, NEJM Journal Watch, Science X Newsletter, and The Scientist. In addition, PubMed was searched using the MESH term “Artificial intelligence”, which yielded 3497 results, of which 2897 pertained to human findings. Further searches yielded 63 clinical studies, 27 studies listed as clinical trials, 72 comparative studies, 70 editorials, 7 evaluation studies, 72 letters, 28 observational studies, 358 reviews, and 50 validation studies. These combined efforts resulted in the identification of 254 original papers published in 2024 that were focused on AI in medical topics. Of these, 83 articles provided information for this review because they presented evidence-based findings. Interpretations of these findings by the authors that published them became the basis for the present narrative review.

## 3. Results

### 3.1. Evaluations

The aim of this review is to create a narrative conversation that provides some insight into the appropriate evaluation of recent AI models used for clinical applications. Many factors are related to the evaluation of AI based on machine learning (ML), a subfield of AI where computers learn and recognize patterns from sets of examples, rather than being programmed with specific rules. Deep learning is a current widely used ML technique that is based on neural network technology with an architecture that tries to parallel the human brain to learn to recognize complex patterns. With this in mind, research efforts need to develop insights to understand why any AI system behaves a certain way or produces a particular result. Some insights into AI performance can be gained by considering that the data in a training model may be inadequate or imperfect. It is important to note that AI capable of addressing varied and complex problems on par with human intelligence is still a long way off. For these reasons, ongoing evaluations of results produced by AI systems are necessary.

One effective test of AI performance is to specifically check the AI’s ability to provide exact and real answers to queries that can be verified in the published literature. The following example is one that is pertinent to medical practitioners using AI for practice guidance: In November 2023, Bard was asked for citations for the information that it provided for use in a continuing medical education course. In checking for accuracy, the first citation provided on the journal’s website was not able to be located, and this was also the case when using the DOI (digital object identifier), which returned the message “DOI not found” [13]. Repeated querying returned the original response. Finally, the chatbot was asked “Does this paper actually exist?” and then replied “I apologize for the mistake. I have double-checked, and the paper does not exist. I am still under development, and I am always learning. I will try my best to avoid making similar mistakes in the future”. In another documented case, it was reported that 70% of the cited references produced by a chatbot were inaccurate [14]. In these examples, the responses may have been overly governed more by creative style rather than by extracting exact information, resulting in what is widely recognized as “hallucination”. Limitations in the degree to which AI tools are trained on or can access medical datasets can contribute to the likelihood of the AI producing false human-like content and hallucination results [15]. Despite this being a well-known complication of narrative AI, users may not be aware of when it occurs. This example highlights the reality that the reliability of the answers provided by this rapidly emerging technology can be questionable. A generative AI model (GPT-4) performed similarly to other modern differential diagnosis generators, achieving a correct most likely diagnosis in only 39% of cases [16], with the authors feeling that it performed as a “black box”. Advocates for utilizing large language models in academic surgery have been open to exploring utilizations but recognize the need for validating content [17].

To ensure that use of AI in American healthcare is fair, appropriate, valid, effective, and safe, some have proposed establishing public–private partnerships to create nationwide health AI assurance labs in which best practices concepts could be applied for testing AI models. Reports on performance could be widely shared for managing AI models over time, as well as over populations and sites where various AI models are deployed [18].

### 3.2. Gynecologic Oncology

There are many potential utilizations of AI in gynecologic oncology, as the specialty spans cancer diagnostics and treatment. Gynecologic cancer diagnosis often requires expert physical exams, radiologic studies, and pathology review, each of which has been considered for AI utilizations. Narrative AI in gynecologic oncology has been explored to evaluate clinical plans, as well as radiologic and pathologic results. In a structured examination in Obstetrics and Gynecology, spanning seven sections, ChatGPT was reported to score 3.5% higher than an average historic human score, while in the gynecologic oncology section, ChatGPT scored 92% against passing at 71% and a human score of 76.9% [19]. While AI may score higher on knowledge-based exams, physicians provided higher-quality responses to gynecologic oncology clinical questions compared to the chatbot. In providing responses to ten questions about the nuanced knowledge and management of gynecologic cancers, physicians were more accurate (86.7%) than ChatGPT (60%) and Bard (43%; *p* < 0.001 for both) [20]. Importantly, physician responses were judged to be best in 76.7% of evaluations versus ChatGPT (10.0%) and Bard (13.3%; *p* < 0.001). Preliminary work has been reported that describes a machine-learning model based on clinical characteristics and qualitative radiological sonographic indicators operating as a waterfall classification model (benign tumor, invasive cancer, or ovarian metastasis) for characterizing solid adnexal structures, operating with an accuracy of 86.4%, sensitivity of 93.8%, and specificity of 86.96% and distinguishing the benign from the malignant 90.91% of the time and the nonmetastatic from the metastatic 91.4% of the time [21]. Importantly, this system includes a tool to identify the contribution of each feature to the classification outcome generated by the machine learning model, thereby providing the clinician with a means for understanding how the automated system arrived at a given decision. An automatic approach of sonogram analysis based on a deep convolutional neural network (ConvNeXt-Tiny) showed robust and stable performance in classifying ultrasound images in the O-RADS v2022 (categories 2–5) radiology reporting system, with an accuracy of 85.9% [22]. A recent systematic review explored the role of AI in ultrasound imaging in gynecologic oncology [23] and identified the aggregate shortcomings of published studies as having high risks of bias for subject selection (i.e., sample size, source, or unspecified scanner model, as well as data not derived from open-source datasets, lack of imaging preprocessing, etc.) and AI models that were not externally validated. However, it was felt that the current evidence supports the role of AI as a complementary clinical and research tool in diagnosis, patient stratification, and prediction of histopathological correlation in gynecological malignancies. These efforts concluded that AI models will have the ability to discriminate between benign and malignant ovarian masses. With these issues in mind, a recent systematic review and meta-analysis whittled down 3726 potential papers for inclusion to 14 papers, 8 of which utilized machine learning, while 6 employed deep learning. They found that there were wide ranges in sensitivity (0.4 to 0.99) and specificity (0.82–0.99) in ovarian cancer diagnosis [24]. This report concluded that overall sensitivity was 81% (95% CI, 0.80–0.82), and specificity was 92% (95% CI, 0.92–0.93), indicating, from this global assessment, that AI “demonstrates good performance in ultrasound diagnoses of ovarian cancer”. However, it did state that “Further prospective work is required to further validate AI for its use in clinical practice”. Another preliminary investigation used machine learning to integrate an automated network for MRI image segmentation in combination with multiple conventional test indicators. This approach achieved an overall sensitivity of 91.9% and specificity of 86.9% in the detection of ovarian cancer that appeared independent of stage, with sensitivities for the detection of early- and advanced-stage ovarian cancer being similar [25]. In addition to radiological uses, machine learning has also been studied with regard to pathology and visual screening. Utilization of a deep learning model, designed to automatically detect serous tubal intraepithelial carcinoma (STIC), the precursor lesion to high-grade serous ovarian carcinoma found in the fallopian tube, was reported to demonstrate increased accuracy and sensitivity with a significant reduction in slide review time when evaluated by a diverse group of pathologists from 11 countries [26]. Another deep learning model has been reported to predict homologous recombination deficiency (HRD) from H&E slides so that breast and ovarian cancers can be treated with poly(ADP-ribose) polymerase inhibitors without the need for expensive next-generation sequencing [27]. A recent study reported enhancing the diagnostic accuracy of transvaginal ultrasound for distinguishing endometrial cancer and endometrial atypical hyperplasia through the integration of artificial intelligence in women with postmenopausal bleeding, achieving high sensitivity (0.87) and specificity (0.86) using an automated segmentation approach [28]. In cervical cancer screening, there have been a plethora of reports using cervical cancer images to predict cervical cancer with outstanding accuracy, coupled with a subsequent disappointing lack of confirmation [29,30,31,32,33].

Finally, efforts integrating ChatGPT treatment recommendations with those from an institutional molecular tumor board resulted in 45.5% agreement with the molecular tumor board on 114 cases involving endometrial/uterine, ovarian, vulvar, cervical, and undefined gynecologic cancers [34].

### 3.3. Clinical Medicine in General

In addition to gynecologic oncology, AI has also been adapted in various clinical applications throughout other fields of medicine. Narrative AI was heralded initially, and within a year, various capabilities and improvements were promoted; however, there have been published reports questioning chatbot accuracy. ChatGPT achieved an accuracy of only 60.3% in forming accurate initial differential diagnoses, and its performance was described as inferior [35]. In another report, Chatbot GPT4 performed similarly to physicians and outperformed residents with regard to clinical reasoning outcomes [36]. ChatGPT-3.5 turbo-0301 significantly underperformed at providing accurate cancer treatment recommendations and generated narratives that were discordant with NCCN recommendations a third of the time [37]. Similarly, an investigation on the quality of information and misinformation about skin, lung, breast, colorectal, and prostate cancers provided by four AI chatbots (ChatGPT version 3.5, Perplexity (Perplexity.AI, San Francisco, CA, USA), Chatsonic (Writesonic, San Francisco, CA, USA), and Bing AI (Microsoft, Redmond, WA, USA)) concluded that the limitations observed suggest that AI chatbots should be used as a supplementary source, rather than a primary source, for medical information [38]. Faced with answering “Questions to Ask About Your Cancer”, recommended by the American Cancer Society, both ChatGPT-3.5 and Bing responded correctly in less than 80% of cases [39]. ChatGPT-4 correctly diagnosed only 57% of complex clinical cases [40].

AI also had mixed performance on board-style examinations compared to humans. When presented with a board-style neurology examination, ChatGPT4 correctly answered 85% correctly on over 1900 questions in behavior-, perception-, and psychology-related areas, using confident language for both correct and incorrect answers [41]. A study on responses to subspecialty questions regarding nephrology by several LLM AIs (Llama2-70B, Koala 7B, Falcon 7B, Stable-Vicuna 13B, and Orca-Mini 13B utilizing GPT-4 and Claude 2) concluded that, overall, 30.6% of 858 multiple choice questions in the Nephrology Self-Assessment Program were correctly answered, although Claude 2 (54.4%) and GPT-4 (73.3%) performed better [42]. However, an Israeli study compared ChatGPT-3.5 and ChatGPT-4 in head-to-head competition with 849 practicing physicians on the 2022 Israeli board residency exams and found that ChatGPT4 passed board residency exams in four out of five specialties with a score higher than the official passing score of 65% while outperforming a considerable fraction of practicing physicians [43]. In contrast, GPT-3.5 did not pass the board exam in any discipline and was inferior to the majority of physicians in the five disciplines. Using AI guidance in clinical practice can increase risk of errors. In a trial involving 457 clinicians who were randomized to study six vignettes using AI model input with or without AI model explanations (among these six vignettes, three vignettes included standard-model predictions, and three vignettes included systematically biased model predictions), physician accuracy was 73% alone and increased by just 4.4% with AI explanations [44]. However, the systematically biased AI model information decreased clinician accuracy by 11.3 percentage points. Thus, there is an inherent risk that errant information provided by AI can negatively affect medical decision making by clinicians. In another report on responses by GPT-4V, Gemini Pro, and four language-only models—GPT-4, GPT-3.5, and two open-source models, Llama 2 (Meta) and Med42—to clinical vignette questions that included both case descriptions and medical imaging, all LLMs were less accurate than human responders and displayed a range of accuracies (44.1–88.7%), with GPT-4 having the best performance [45]. Comparative evaluations of LLMs (GPT-3.5, GPT-4, PaLM 2, Claude-v1, and LLaMA 1) on 2044 clinical oncology questions also yielded variable accuracies (25.6–68.7%), with random guess correctness estimated at 25.2%, leading to the conclusion that there are models that appear to perform no better than random chance, whereas others may achieve a level of accuracy competitive with resident physicians [46]. Using additional LLMs (ChatGPT-3.5, ChatGPT-4, Mistral-7B-Instruct-v0.2, Mixtral-8x7B-v0.1, Llama-2-13b-chat, Nous-Hermes-Llama2-70b, openchat-3.5-1210, BioMistral-7B DARE) on medical oncology examination questions, and extending evaluations to three multimodal and seven unimodal text-only chatbots (ChatGPT-4 Vision, Claude-3 Sonnet Vision, Gemini Vision, ChatGPT-3.5, ChatGPT-4, Claude-2.1, Claude-3 Sonnet, Gemini, Llama2, and Mistral Large), similar results were reported [47,48]. In addition, ranges of performance of these same LLMs have been reported for clinical knowledge and reasoning in ophthalmology [49]. The use of AI has also been explored in day-to-day clinical tasks, such as documentation and patient communication. However, physicians were not more efficient when using voice-enabled AI to automatically document conversations between physicians, patients, and their families, intended to make it possible for physicians to give their full attention to the patient while AI technology created complete, accurate clinical notes directly in the electronic hospital record for the clinician to review and sign (ambient clinical documentation via DAX Copilot) [50]. A report that considered generative AI-drafted replies for patient messages reported increased physician time spent on answering messages. There was a significant increase in read time, no change in reply time, and significantly longer replies. Increased read time seemed to be attributable to the need to read both the patient’s original message and the draft reply in order to avoid AI hallucinations [51]. Similarly, a significant subset of clinicians using an AI-powered clinical documentation tool did not find time-saving benefits or improved electronic health record experiences [52]. Answers by ChatGPT have previously been reported to be ~4X longer in length, but the chatbot responses were rated to be of significantly higher quality (3.6X) and demonstrate ~10X more empathy than those of physicians [53,54]. In a study of 1600 emergency medicine patient medical records, LLM-generated emergency medicine-to-inpatient handoff notes were assessed as superior to physician-written summaries but marginally inferior in usefulness and safety [55]. AI has also been investigated in extracting information from the patient’s medical record. A utilization of large language model AI (LLM AI) with wide appeal is the extraction of unstructured data from clinical notes in electronic medical records. In a recent report, highly detailed prompts from the LLM AI (ChatGPT-4) were needed for agreement with text string searches; however, the retest reliability observed that the LLM was consistent in misclassification-type hallucinations and did not perfectly replicate its findings across new session queries on 5 different days [56]. Thus, the application of LLMs to data extraction from electronic clinical notes can accomplish considerable efficiency that is accompanied by trade-offs in accuracy, problems in interpretation by AI (“unhealed”, “w/o healing”, “no healing”), and ambiguous terms (“S/P healing”). It is apparent at present that LLM performance requires meticulous engineering of the prompt syntax methods for understanding nuanced clinical language [57]. Other investigators reported that there was no change in reply action time, write time, or read time in the application of AI-generated draft replies to patient portal messaging [58]. Of note, an evaluation of GPT-3.5, GPT-4, and Claude AI found that Claude AI provided the best overall measures of quality, empathy, and readability, with results comparable to responses from physicians with respect to drafts of responses to cancer patient questions [59]. Similar findings were reported for ChatGPT 3.5 in terms of correctness, conciseness, completeness, and potential for harm, with the chatbot scoring 30% higher than human experts on readability of AI-generated responses to questions in radiation oncology [60]. In the medical specialty of radiology, attempts have been made to implement AI to recognize specific findings in images. A report on lower-performing radiologists challenged the notion that AI assistance would increase their performance, finding that AI errors adversely influenced radiologist performance and treatment outcomes [61]. An encouraging proof of concept has been reported for Med-PaLM Multimodal as a generalist AI system that interprets clinical terms, imaging, and genomics using zero-shot clinical reasoning, to the extent that four clinicians preferred reports generated by this AI system over those produced by radiologists in 40.05% of 246 radiology case evaluations, with error rates similar to human radiologists [62]. The reader of this report will notice that the end results are quite nuanced across model sizes and presented more from the standpoint of computer scientists than clinicians. Similarly, it has been reported that the availability of ChatGPT Plus [GPT-4] to physicians as a diagnostic aid did not significantly improve clinical reasoning over conventional resources (UpToDate and/or Google). Importantly, the LLM alone demonstrated higher performance than the physician group using GPT-4 or the physician group using conventional resources, revealing a need for physician development to achieve elevated performance through physician–artificial intelligence collaboration in clinical practice [63]. Recently, the FDA authorized Sepsis ImmunoScore, an AI-based software designed to identify patients at risk of sepsis that has the potential to identify both sepsis disease risk and sepsis mortality [64]. When queried on the energy content of 222 food items, both ChatGPT-3.5 and ChatGPT-4 provided accurate answers less than half of the time [65].

Histology and pathology have also been recognized as having potential for AI use in clinical medicine. Artificial intelligence of the nucleus based on a deep learning method has been reported to identify specific nuclear signatures at nanoscale resolution based on the spatial arrangement of core histone H3, RNA polymerase II or DNA from super-resolution microscopy images and correctly identified human somatic cells, human-induced pluripotent stem cells, very early-stage infected cells transduced with DNA herpes simplex virus type 1, and even cancer cells [66].

Existing AI methods for histopathology image analyses have been limited to optimizing specialized models for each diagnostic task [67,68]. Although such methods have achieved some success, they often have limited generalizability to images obtained through different digitization protocols or samples collected from different populations [69]. When Bard and GPT-4 were tasked with interpreting 1134 pathology reports for patients, both generated reports that were significantly easier to read; however, GPT-4 interpreted reports correctly 10% better than Bard and had hallucinations that were 10% of those made by Bard [70]. As such, these types of AI interpretations need to be subject to review by clinicians prior to being made available to patients. However, a recent report described a model that was found effective for 19 anatomical sites in samples from diverse populations and processed by different slide preparation methods [71]. This system provides hope when taken in the context of a reported commercial AI algorithm developed for breast cancer detection (INSIGHT MMG, version 1.1.7.2) claiming to identify women 4–6 years prior to eventual detection in retrospective mammograms and thereby offering a pathway that can lead to earlier breast cancer diagnosis [72]. Similarly, the identification of breast cancer relapses in the text of unstructured computed tomography (CT) reports using natural language processing (BlueBERT) led to an accuracy of 93.6%, a sensitivity of 70.1%, and a specificity of 95.3% for regional relapses and an accuracy of 88.1%, a sensitivity of 91.8%, and a specificity of 83.7% for distant relapses [73]. When ChatGPT-4 was used to interpret clinical ophthalmic images, it accurately answered two-thirds of multiple-choice questions that required interpretations of ophthalmic images [74]. However, performance was better on questions unrelated to images than on image-based questions (82% vs. 65%).

Attempts to utilize LLM AI for decisions that impact medical systems have been investigated. ChatGPT-4 Turbo was used for evaluating surgical risk stratification and postoperative outcomes. This AI was able to predict physical status, hospital admission, intensive care unit (ICU) admission, unplanned admission, hospital mortality, and postanesthesia care fairly well; however, predictions of the durations of postoperative issues were universally poor, especially those made by the large language model for PACU phase 1 duration, hospital duration, and ICU duration prediction [75]. AI using policy learning trees tested an approach which can be linked to an electronic health record data platform to improve real-time allocation of scarce treatments [76]. By learning from multiple molecular tumor boards about biomarkers with low evidence levels, an AI system was reported to have a post-training concordance of 88% with molecular tumor boards [77]. ChatGPT-4 has been explored in evaluations of hospital emergency department triage, which involves prioritizing patients based on both the severity of their condition and their expected resource needs, retrospectively using 10,000 patient pairs to find that GPT-4 correctly identified the individual with the higher acuity with an accuracy of 89%, which was similar to the accuracy of physician reviewers [78]. Finally, insurance billing and coding is an additional way in which AI can be used. A machine learning AI algorithm utilizing diagnostic item categories and diagnostic cost group methods has been reported to reliably price even rare diseases, avoiding serious underpayments even for the 3% of people who have at least one diagnosis as rare as one in one million [79]. Importantly, this AI approach can circumvent diagnostic vagueness and attempts to game the payer system. However, GPT-3.5, GPT-4, Gemini Pro, and Llama2-70b have been reported to be poor medical coders in using ICD-9-CM, ICD-10-CM, and CPT code descriptions to generate appropriately specific billings, with GPT-4 having the highest exact match rate (45.9% on ICD-9-CM, 33.9% on ICD-10-CM, and 49.8% on CPT), but often generating codes conveying imprecise or fabricated information [80].

## 4. Discussion

Utilizations of AI in clinical applications fall short of being perfect. The main questions that arise are as follows: (1) How can AI performance be improved? (2) At what level of imperfection can AI be useful clinically? (3) How can imperfections be disclosed to caregivers and patients? It has been the position of physicians that patients should consent to AI use and that patients should have opportunities to choose between physician- and AI-recommended treatment plans [81]. These physician-based perspectives highlight the need for rigorous assessments of how AI impacts clinical care, along with clearly defined accountability for decision making when issues arise from AI utilization. Key concerns among physicians include ethical considerations, such as explainability, patient consent, and responsibility, all of which are essential for the optimal adoption of AI into clinical care. Similarly, 62.7% of people surveyed in the US stated that it was very true that they want to be notified if AI would be involved in their care, while less than 5% answered that they did not find notification important [82].

Issues of AI accuracy have been outlined here in this review of evidence-based reports. A recent search in Copilot ends with the following statement: “AI-generated content may be incorrect”. In essence, this is to say that this AI exhibits the Dunning–Kruger effect by demonstrating unconscious incompetence (i.e., not knowing what it does not know). It is important that users and adopters of AI are also sensitive to the ultracrepidarian identity of AI, which tempts users to believe that an AI application is an expert in all fields or medical specialties (an “everythingologist”), even though its expertise is limited to only certain areas. The old Russian phrase (“*Doveryai*, *No Proveryai*”) meaning “*trust but verify*”, quoted by Ronald Reagan [83,84], certainly has application to AI, but it raises the question of how we can ensure verification. Will there be efforts in place to identify AI hallucination or confabulation? Indeed, how is accountability to be positioned? To what extent is the AI creator/vendor, hospital, clinic, or performing physician responsible for inaccurate or faulty results from an AI that has been utilized? It should not be ignored that artificial intelligence can generate or be intentionally used for prevarication, misinformation, or targeted disinformation. The ability of generative AI to rapidly generate diverse and large amounts of convincing disinformation about vaccination and vaping has been reported to be profound, especially when operation is allowed with few or no guiderails in place [85]. Such results, when targeted to blogs or social media postings, can amount to intentional Weapons of Mass Disinformation replete with scientific-looking reference citations. In these instances, there must be tools in place for fact checking the language content of information originating from generative AI. Lastly, in an AI-dominated clinical world, will human clinical skills be subject to atrophy? Will this possibility have a negative effect on both clinical training and clinical research? The ideas considered here are current to the early part of 2025. It is entirely possible that developments in the near future will address the issues raised here. The AI genie has indeed left the bottle. The degree to which our wishes are granted by the AI genie must be examined.

## 5. Conclusions

This review has considered the range of results from various AIs, which, in some cases, have been poor and underperformed compared with results from trained clinicians. While AI may show mixed success at answering board questions, responses to patients’ questions can often be incorrect. AI generation of clinical notes does not seem to improve efficiency. When AI was combined with physician diagnostics in radiology, the AI negatively influenced physician decision making. While AI usage may have potential for application in radiology, treatment matching, and pathology, expensive and diverse learning is required for accuracy. The current expectation for AI to perform as medical coders for billing purposes has been quite imprecise and led to the inclusion of fabrications. Thus, the consequences of clinical AI utilization can include benefits, inaccuracies, and expectations that are as yet unmet.

## Data Availability

No new data were created or analyzed in this study.
